# Non-nuclear AR Signaling in Prostate Cancer

**DOI:** 10.3389/fchem.2019.00651

**Published:** 2019-09-26

**Authors:** Alice Zamagni, Michela Cortesi, Michele Zanoni, Anna Tesei

**Affiliations:** Biosciences Laboratory, Istituto Scientifico Romagnolo per lo Studio e la Cura dei Tumori (IRST), IRCCS, Meldola, Italy

**Keywords:** prostate cancer, tumor microenvironment, androgen receptor, non-genomic functions, invasiveness

## Abstract

Despite the key role played by androgen receptor (AR) in tumor cell aggressiveness and prostate cancer (PCa) progression, its function in the tumor microenvironment (TME) is still controversial. Increasing studies highlight the crucial role played by TME modulation in treatment outcome and tumor cell spreading. In this context, targeting specific constituents of the TME could be considered an alternative approach to classic treatments directed against cancer cells. Currently, androgen deprivation therapy (ADT) is a routinely adopted strategy in the management of PCa, with initial success, and consecutive fail. A possible justification to this is the fact that ADT aims to target all the transcription/translation-related activities of AR, which are typical of tumor epithelial cells. Less is still known about side effects of ADT on TME. Cancer Associated Fibroblasts (CAFs), for example, express a classic AR, mostly confined in the extra-nuclear portion of the cell. In CAFs ADT exerts a plethora of non-transcriptional effects, depending by the protein partner linked to AR, leading to cell migration, proliferation, and differentiation. In recent years, substantial progress in the structure-function relationships of AR, identification of its binding partners and function of protein complexes including AR have improved our knowledge of its signaling axis. Important AR non-genomic effects and lots of its cytoplasmatic binding partners have been described, pointing out a fine control of AR non-genomic pathways. Accordingly, new AR inhibitors have been designed and are currently under investigation. Prompt development of new approaches to target AR or block recruitment of its signaling effectors, or co-activators, is urgently needed. The present review takes an in-depth look at current literature, furnishing an exhaustive state-of-the-art overview of the non-genomic role of AR in PCa, with particular emphasis on its involvement in TME biology.

## Introduction

Prostate cancer (PCa) is one of the leading causes of cancer death in men, with ~307,000 deaths representing 6.6% of male cancer mortality worldwide (Taitt, 2018). The role played by the androgen receptor (AR) in the development and progression of PCa has resulted in widespread interest in this nuclear receptor. Indeed, AR, a ligand-activated intracellular transcription factor belonging to the steroid hormone receptor family, currently represents the main validated drug target in the management and progression of this neoplastic disease. The identification of the three-dimensional crystal structures of the AR DNA binding domain (DBD) and ligand binding domain (LBD) has increased our understanding of this receptor by bringing to light specific molecular details, mainly about its genomic functions. These findings have been exploited to develop several predictive binding models of novel compounds and to facilitate rational drug design in order to enhance existing drugs and develop new molecules for PCa therapy. However, despite increasingly effective anti-androgen therapies, most patients eventually relapse, developing lethal metastatic castration-resistant prostate cancer (CRPC; Hotte et al., 2018). In particular, for this subtype of patients there is a huge need of innovative therapeutic approaches.

Notably, although the genomic function of AR is considered to be the main role of this nuclear receptor, lots of binding partners of AR have been described in the cytoplasm highlighting a fine tuning of AR non-genomic pathways whose involvement in prostate cancer disease and progression is currently subject to debate.

The present review aims to provide state-of-the-art knowledge on the most important non-genomic effects of AR receptor and the compelling evidence underlying their potential involvement in prostate cancer are here discussed.

### The Androgen Receptor and Its Canonical Genomic Activity

**Figure 1:**
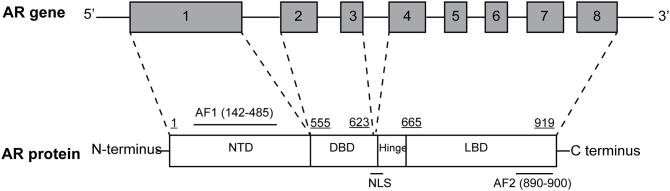
Structural organization of the full-length androgen receptor (AR) gene and protein. AR gene contains eight exons: exon 1 encodes for N-terminal domain (NTD), containing the AF1 transactivation function; exon 2 and 3 encode for DNA-binding domain (DBD); exons 4–8 encode for C-terminal ligand-binding domain (LBD), containing the AF2 transactivation function. The hinge region, containing the nuclear localization signal is encoded by the 5′ region of exon 4.

The AR gene, located on the long arm of the X-chromosome (locus: Xq11-q12), comprises eight exons interrupted by introns of different lengths (0.7–2.6 kb) and encodes a 110-kDa protein made up of 919 amino acids (Figure 1). AR shares with the other members of steroid hormone receptors superfamily—including estrogen, progesterone, glucocorticoid, and mineralcorticoid receptors—a modular structure consisting of three major functional domains: N-terminal domain (NTD) encoded by exon 1, DNA-binding domain (DBD) encoded by exons 2 and 3, and C-terminal ligand-binding domain (LBD; McEwan, 2004) encoded by exons 4–8.

The LBD is the most widely studied and exploited AR domain by Big Pharma due to its crucial role in ligand binding and consequent AR activation. It is constituted by 11 α-helices (α-helix 2 is lacking) and two short β-turn strands arranged in three layers to form an antiparallel “α-helical sandwich.” In particular, upon agonist binding, α-helix 12 (H12) is repositioned and serves as the “lid.” LBD forms the second β-turn that serves as a “lock” to better stabilize the “lid.” A plethora of molecules have been designed taking into account H12, because of its crucial position and pivotal role in AR action mechanism (Tran et al., 2009; Clegg et al., 2012; Balbas et al., 2013; Guerrini et al., 2014).

Two transactivation domains within NTD and LBD have also been identified: constitutively active activation function 1 (AF1) and ligand-binding dependent activation function 2 (AF2). Both domains bind specific co-regulatory proteins and are crucial for the activity of the full-length receptor, as they are also used for drug development and therapeutic targeting of PCa (Bevan et al., 1999; Kumar, 2016). Moreover, a small portion of molecule between DBD and LBD termed “hinge region” have been identified (Figure 1) and considered as a potential therapeutic target. In particular, the (629)RKLKKL(634) motif of this region is key in controlling AR activity, serving as the main part of the nuclear translocation signal and regulating the transactivation potential and intranuclear mobility of the receptor. In addition, it is a target site for acetylation, ubiquitylation, and methylation (Clinckemalie et al., 2012).

Unliganded AR is sequestered in the cytoplasm by a chaperone complex including Hsp90 and interacting with LBD (Pratt et al., 1989). Upon androgen stimulation through dihydrotestosterone (DHT) or testosterone (T) binding to LBD, AR changes its conformation, dissociating from the chaperone complex, dimerizing, and translocating into the nucleus. Here, AR impacts gene expression by binding specific DNA motifs localized in the regulatory regions of its target genes. In particular, it is known that AR functionally interacts with two different sets of AREs: cAREs (classic steroid-hormone-response elements), which are three-nucleotide-spaced partial-palindromic repeats of the AR monomer consensus binding site 5′-TGTTCT-3′, and sAREs (selective AREs) which are three-nucleotide-spaced partial direct repeats of the same monomer-binding element and_resemble the binding. Once bound AREs, AR recruits co-activators or co-repressors to regulate gene expression (Shang et al., 2002). Widespread interest in AR co-regulatory research has also been promoted by the fact that new therapeutic methods could be developed on the basis of the presence of specific AR co-repressor mutations or altered expression, correlated with specific diseases. Thus, co-regulators have also aroused interest as potential targets for drug treatments or diagnostic markers for PCa (Wang et al., 2005).

### LBD as a Therapeutic Target for CRPC

First-generation antiandrogens such as bicalutamide or flutamide have shown good ability in AR-LBD binding, preventing androgen's occupation in AR pocket. Unfortunately, they failed to demonstrate efficacy in CRPC patients. For this reason, improved second-generation molecules have been designed and are currently in clinic.

**Figure 2:**
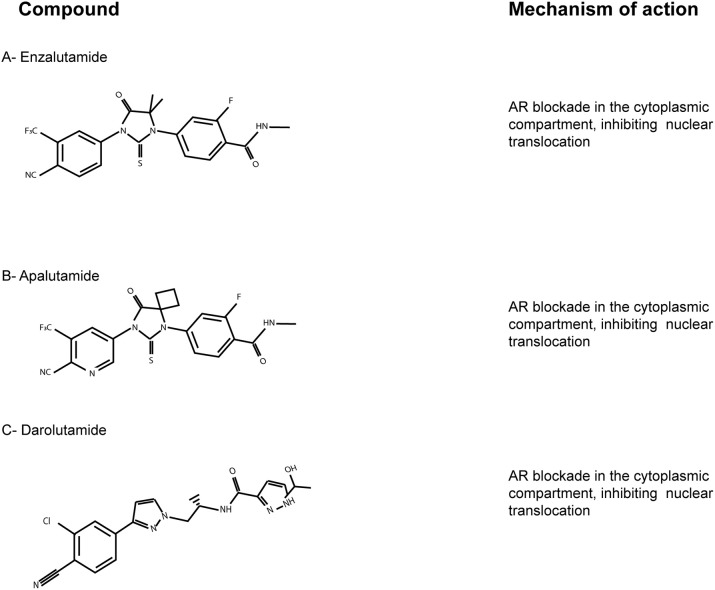
The AR-LBD as a therapeutic target. Chemical structures of the main AR-LBD molecules currently in use for CRPC patients. Main mechanisms of inhibition are reported. **(A)** Enzalutamide. **(B)** Apalutamide. **(C)** Darolutamide.

Enzalutamide (Figure 2A) has greater affinity for AR-LBD than bicalutamide, with an additional strong effect on AR mutant W741C (Tran et al., 2009). In the PROSPER phase III trial (NCT02003924) enzalutamide-based therapy led to a striking 71% lower risk of metastasis and death, compared to placebo, among men with nonmetastatic CRPC (Hussain et al., 2018).

Apalutamide (ARN-509; Figure 2B) is an AR targeted antiandrogen with a chemical structure very similar to enzalutamide, but characterized by a better affinity to AR-LBD. It lacks agonist activity, in contrast with bicalutamide, and inhibits nuclear translocation and DNA binding (Clegg et al., 2012). Moreover, apalutamide showed less blood-brain barrier penetration in murine xenograft models of CRPC than enzalutamide, which might lead to less seizure than enzalutamide-based therapies. On the basis of the SPARTAN trial (NCT0946204), apalutamide was FDA-approved in 2018 for patients with nonmetastatic CRPC (Smith et al., 2018).

Lastly, darolutamide (ODM-201; Figure 2C) is an antiandrogen with higher potency and efficacy toward AR-LBD compared to enzalutamide and apalutamide, but very similar mechanism and pharmacology. The results of the phase III ARAMIS trials (NCT02200614) have just been published, suggesting this new AR inhibitor as an alternative option for nonmetastatic CRPC patients (Burki, 2019). ARAMIS results are indeed in line with those of the SPARTAN and the PROSPER trials, involving apalutamide and enzalutamide, respectively. Since darolutamide has a different molecule structure, it has to be taken into account that it may be related to different adverse effects. Thus, further observations on real-world data are needed to better define the clinical niche for each compound (Higano, 2019).

## AR Non-genomic Signaling in Prostate Cancer Cells

In the past, interactions with AR followed by transcription factor activity were indicated as the main molecular mechanisms responsible for androgen activity. However, there is growing evidence that many cellular responses to androgens can also act through mechanisms that are independent of the ligand-dependent transactivation function of AR. This is called “non-genomic” signaling and typically occurs within a short time frame (i.e., seconds to minutes). It is not weakened by transcription inhibitors and does not require functional nucleus or transcription/translation machinery activation (Losel et al., 2003; Foradori et al., 2008).

Below the major signal transduction pathways activated by rapid, non-genomic AR signaling will be reviewed by looking in depth at the implications for current and novel therapies targeting different AR domains.

### AR and Src

**Figure 3:**
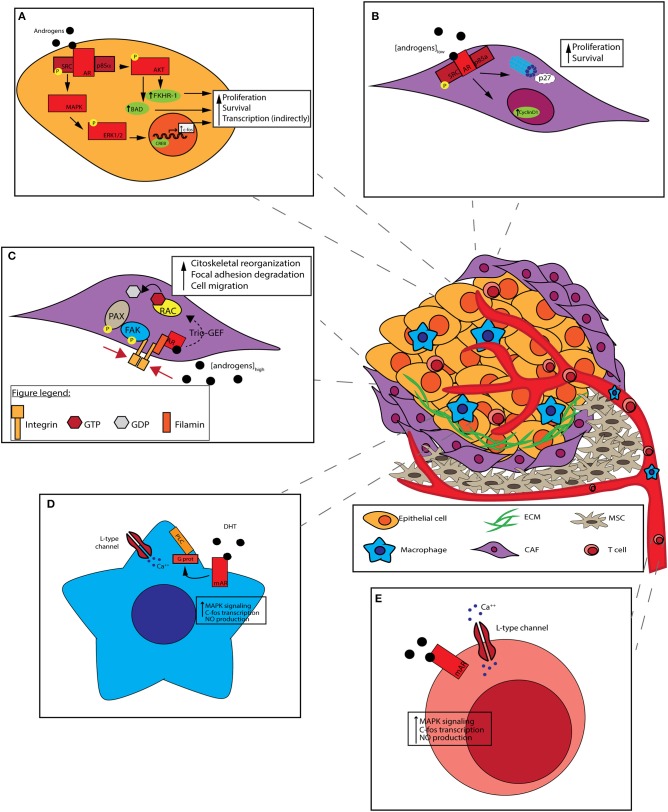
Non-genomic effect of AR on prostate cancer cells and tumor microenvironment (TME). Prostate cancer cells interact with heterogeneous cell populations composing TME: the recruitment of mesenchymal stem cells (MSCs) and the activation of cancer-associated fibroblasts (CAFs) are due to molecules secreted by tumors cells themselves along with the formation of aberrant vasculature. T cells and other immune cells are attracted by various chemokines and cytokines to the tumor. TME architecture can influence tumor features. **(A)** c-SRC activation by non-genomic signaling of the AR. AR binds to the Src, enabling its autophosphorylation. Activated Src trigger cell cycle progression, through the MAPK/ERK/CREB cascade. CREB activation induces the expression of different genes, like c-FOS. PI3K activation by non-genomic signaling of the AR by its binding to PI3K regulatory subunit p85α. PI3K activation leads to AKT phosphorilation which activates Bad and FKHR-1. Both non-genomic AR signalings result in cancer cells survival promotion. **(B)** In CAFs, androgens stimulate DNA synthesis at pM (low) concentrations: stromal AR associates to Src and p85α, leading to cyclin D1 upregulation and p27 degradation, thus promoting cell proliferation. **(C)** In CAFs, androgens stimulate cell motility at nM (high) concentrations: stromal AR associates to Filamin A, forming a complex including also integrin beta 1. The complex co-localizes with intermediate cytoskeletal filaments, promoting FAK and PAX phosphorylation, and Rac activation. Both pathways result in cell migration promotion. **(D)** Macrophages lack of classical intracellular AR. DHT stimulation occur through mAR which is coupled to phospholipase C (PLC) via a pertussis toxin-sensitive G-protein. After the binding of DHT a rapid increase in intracellular free [Ca^++^] is mediated, leading to MAPK cascade activation, c-FOS transcription, and NO production. **(E)** In T cells, androgens stimulation occur through mAR. T cells respond to low androgen concentrations with a rapid rise of [Ca^++^] with a mechanism similar to that of macrophages.

It is known that c-Src, a non-receptor tyrosine kinase, regulates a complex signaling network driving the development of castration-resistance and bone metastases, both of which indicate the lethal phenotype of advanced disease. Preclinical studies have confirmed a role for c-Src and Src family kinases (SFKs) in proliferation, angiogenesis, invasion and bone metabolism (Thomas and Brugge, 1997; Marzia et al., 2000; Miyazaki et al., 2004; Roskoski, 2004; Schenone et al., 2007; Guarino, 2010), indicating the involvement of Src signaling in both epithelial and stromal mechanisms of disease progression. It has been demonstrated that c-SRC may be activated by rapid, non-genomic AR signaling (Fizazi, 2007; Figure 3A). In particular, AR enhances kinase activity by binding a proline-rich sequence present in the NTD to Src homology domain 3 (SH3), thus twisting it from an inactive to active conformation by autophosphorylation of Src kinase domain. After stimulation, Src mediates cell cycle progression through the MAPK/ERK/CREB cascade (Peterziel et al., 1999; Unni et al., 2004; Zarif et al., 2015). Once CREB has been activated, it becomes a transcription factor inducing the expression of several genes, including *c-fos* (De Cesare et al., 1998), highlighting how AR stimulation can mediate gene transcription even in a non-genomic way. Notably, the above-described interaction between the AR NTD polyproline domain and SH3 of Src may be hampered by the use of AR NTD inhibitors (see also section The AR N-Terminus Domain as a Therapeutic Target).

### AR and PI3K

In addition to Src, its main extra-nuclear partner, AR may interact with p85α, the regulatory subunit of PI3K (Baron et al., 2004), a well-known oncogenic protein kinase which plays a pivotal role in malignant transformation, disease progression, and metastatization process (Graupera et al., 2008; Kalaany and Sabatini, 2009; Karki et al., 2016). In particular, a phosphotyrosine-rich region of AR NTD can bind SH2 domain of p85α. This binding results in the activation of the PI3K catalytic subunit, with a consequent production of phosphatidylinoditol-3,4,5-triphosphate (PIP_3_) and downstream phosphorylation of Akt and its targets Bad and FKHR-L1 (Forkhead in rhabdomyosarcoma-L1). As a result, survival pathways are activated with a clear protection against apoptosis by androgens (Yang et al., 2005). Curiously, and in an opposite trend with respect to Src activation, PI3K/Akt pathway activation is not ligand-dependent.

Even though PI3K inhibitors (Sarker et al., 2009) have shown little activity in monotherapy trials for PCa management, their timing and combination with ADT, in light of the reported considerations, could be better explored to offer promising therapeutic approaches.

## AR Non-genomic Signaling in Prostate Cancer-Associated Stromal Cells

### The Role of AR in TME

Normal prostate gland is composed of epithelium-lined prostatic ducts and a stroma composed of smooth muscle cells and a small number of fibroblasts, endothelial cells and nerves (Josson et al., 2010). In prostate cancer, stromal cells develop a progressively altered phenotype, with typical stress-condition features, like extracellular matrix (ECM) remodeling, enhanced angiogenesis, and inflammatory cells infiltration (Rowley, 1999). In this context, crosstalk between epithelial and stromal PCa cells may sustain disease progression by the release of soluble growth factors, cytokines and chemokines, stimulating neo-angiogenesis, and invasion (Karlou et al., 2010).

Numerous studies have looked at the part played by AR in epithelial cells, but very few have focused on its role in stromal counterparts (Leach and Buchanan, 2017). It is established, that various cells of prostate TME express AR and this might affect PCa in different ways. Given the growing importance of TME in PCa development and progression, more research is needed to explore the presence and function of stromal AR, also from a pharmacological point of view.

Interestingly, loss of AR expression in tumor stroma increases the risk of relapse after radical prostatectomy and it is commonly accepted that stromal AR expression progressively decreases during PCa progression, further enabling the growth of carcinoma cells (Henshall et al., 2001; Ricciardelli et al., 2005; Singh et al., 2014). It was recently demonstrated in a transgenic mouse model that spontaneously developed orthotopic PCa that conditional AR knockout in both stromal and epithelial cells resulted in smaller tumors with a less-aggressive signature than AR knockout in epithelium alone, suggesting a possible proliferating role of stromal AR in primary prostate tumor growth (Niu et al., 2008).

Finally, in different cell types, non-genomic androgen actions not modulated by cytoplasmatic AR, have been reported by several authors. In particular, Lang et al. (2013) showed that AR non-genomic responses were mediated by a membrane-embedded receptor called membrane androgen receptor (mAR). mAR is activated when a steroid is conjugated to molecules that cannot enter deep into the cytoplasm or translocate to the nucleus when bound to AR, as in the case of testosterone conjugated with bovine serum albumin (T-BSA). Even if mAR structure has not been identified, four distinct proteins have been proposed as putative mAR: TRPM8, OXER1, GPRC6A, and ZIP9 (Thomas, 2019). Research conducted to date suggests that a large part of non-classic, cell surface-initiated androgen activity is mediated by novel mAR, which is not related to cytoplasmatic AR (Lang et al., 2013), as confirmed by Thomas et al. (2017).

Below an overview on the role of AR in different cells composing TME and its involvement in prostate cancer disease is reported.

### AR and Cancer-Associated Fibroblasts (CAFs)

CAFs are dominant components of PCa stroma. However, the contrasting effects of the androgens on CAFs and adjacent normal prostate fibroblasts (NPFs) are still poorly defined as well as the mechanisms triggered by AR.

In CAFs, androgens would seem to stimulate DNA synthesis at low picomolar concentrations, enhancing cell motility at high nanomolar concentrations. CAFs may thus shift from a proliferative to a migratory phenotype, depending on the androgen concentration present in the medium (Castoria et al., 2014).

This bipartite condition was represented in a preclinical model by Castoria et al. where, when suboptimal (1 pM) concentration of androgens is present in the medium, stromal AR associates to Src and p85α (Castoria et al., 2014). This association enhance MAPK and Akt activity, leading to cyclin D1 upregulation and p27 transport in cytoplasm for degradation. As a result, cell proliferation is fostered (Figure 3B; Castoria et al., 2003). On the contrary, at optimal (10 nM) androgens concentration, stromal AR associates with an actin-binding protein, Filamin A (FlnA), forming a complex including integrin beta 1. AR/FlnA/integrin beta 1 complex co-localizes with intermediate cytoskeletal filaments, and promotes two independent pathways: on the one hand, it fosters FAK and paxillin phosphorylation, on the other hand it activates Rac. Both pathways result in cytoskeleton reorganization, focal adhesion degradation and, as a consequence, cell migration (Figure 3C; Castoria et al., 2011). The ultimate result of enhanced migration is still unclear, but CAFs might move toward PCa epithelial cells, maybe sustaining their invasiveness.

Moreover, it was recently observed that, in mouse embryo-,immortalized-, and transformed- fibroblasts, bicalutamide, a non-steroidal competitor of androgens on AR, inhibits the above described migratory phenotype by preventing the AR/FlnA complex assembly, but supporting the proliferative phenotype (Castoria et al., 2014). Given that after bicalutamide treatment often PCa progression was observed (Vander Griend et al., 2007), these findings highlight how ADT may also bring undesired side effects on stromal cells and that new innovative approaches taking into account these aspects should be developed.

### AR and Macrophages

It has been seen that the majority of neoplastic tissue contains a large number of macrophages (i.e., tumor associated macrophages, TAM) representing the main components of the host leukocytic infiltrate (Van Ravenswaay Claasen et al., 1992). Macrophages recruitment in TME is mediated by a variety of chemoattractants, and exhibit M2-type characteristics (Sica et al., 2006) and promote prostate cancer metastasis (Izumi et al., 2013; Lin et al., 2013; Maolake et al., 2017). Interestingly, in macrophages that lack classic intracellular AR, testosterone stimulation eventually occurs through mAR which is functionally coupled to intracellular [Ca^++^] homeostasis. It has been shown that, in macrophages, mAR is coupled to phospholipase C via a pertussis toxin-sensitive G-protein (Figure 3D); a rapid increase in intracellular free [Ca^++^] is induced after testosterone binding, coupled with inositol 1,4,5-trisphosphate formation (Benten et al., 1999).

An increase in intracellular [Ca^++^] occurs within few seconds, mainly the result of a release of [Ca^++^] from intracellular stores (Benten et al., 1999). If the use of T-BSA or AR antagonists, such as flutamide or cyproterone acetate, preserves the above described pathway, the use of phospholipase C inhibitor (U-73122) and G-protein inhibitor (*pertussis* toxin) ends up complete blocking the mobilization of intracellular [Ca^++^]. Such findings suggest the involvement of mAR in androgen-dependent [Ca^++^] response, involved in the regulation of many cellular mechanisms, including phagocytosis (Nunes and Demaurex, 2010). However, the meaning of such findings, in the context of the progression of neoplastic disease remains to be elucidated.

### AR and T Cells

In T cells a similar mechanism of action involving mAR is proposed. T cells respond to testosterone (T) with a rapid rise of [Ca^++^] due to an enhanced influx of extracellular Ca^++^ channel-mediated (Figure 3E; Wunderlich et al., 2002). Few data are available about the ultimate cellular effect consequent to this phenomenon. Ca^++^ serves as a ubiquitous second messenger molecule, impacting numerous intracellular processes ranging from cell proliferation to apoptosis and motility.

### AR and Endothelial Cells

Endothelial cells, a key component of blood vessels, express AR, but little is known about the function and impact of androgens on these cells (Liu et al., 2003). An interesting study, reported by Yu et al. (2012), emphasizes how testosterone and testosterone bound to albumin (T-BSA) enhance endothelial nitric oxide synthase (eNOS) activity, in a rapid (30 min), non-genomic way, promoting nitric oxide (NO) release from human aortic endothelial cells, via PI3K/Akt pathway. Although further research is needed to elucidate non-genomic AR signaling in endothelial cells, it is remarkable that NO production supports immune suppressive microenvironment, enhancing cancer growth (Grimm et al., 2013). As far as we know, there are no specific studies focusing on PCa, non-genomic AR role and neo-angiogenesis, making it a fascinating research field.

## The AR N-terminus Domain as a Therapeutic Target

As reported above, the AR NTD carries out a plethora of tasks, spanning from the AR transactivation (by the AF1 domain) to the recruitment of cytoplasmic binding partners, which are the topic of the present review. In great contrast with AR LBD, AR NTD is an intrinsically disordered protein domain which lacks of a stable secondary structure; for this reason, it is not easy to target using structure-based drug design. It is characterized by the presence of two large polymorphic glutamine (20–23 residues) and glycine (16–23 residues) stretches, two glutamine repeats (5 and 6 aminoacids), shorts repeats of 5 alanine and 8 proline residues and a repeat of the sequence PSTLTL (Choong et al., 1998; Reid et al., 2002; Betney and McEwan, 2003; Davies et al., 2008).

Bioinformatic and *in vitro* studies have described the AR NTD transactivation domain AF1 as an ensemble of different conformations with limited secondary and tertiary structures, primed to bind co-regulatory partners. After binding with a co-regulatory protein, the percentage of helices in AF1 increases to 35–40%, from an initial 13–16%, leading to the stabilization a particular conformation (McEwan, 2012).

Despite the intrinsic complexity of this domain, AR NTD inhibitors could have the enormous advantage to target also AR splice variants, which keep to be expressed in CRPC patients. In this scenario, potential AR NTD inhibitors may be also employable in all stages of the disease.

Few molecules are at the moment, both in preclinical and clinical development (Antonarakis et al., 2016) and as frequently reported herein, the aim of this review is to arouse the interests of chemists to invest in the design of other AR-NTD inhibitors.

### 3E10-AR441

**Figure 4:**
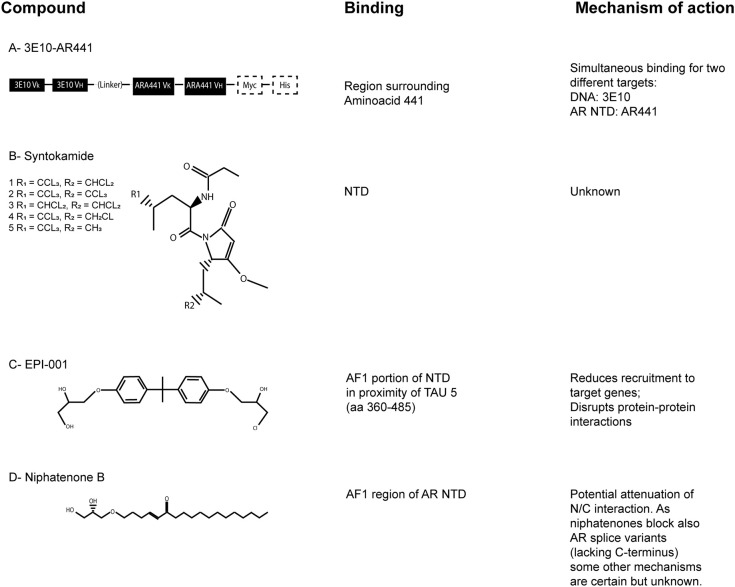
The AR-NTD as a therapeutic target. Chemical structures of the described AR-NTD molecules. The precise binding site and main mechanisms of inhibition are reported. **(A)** 3E10-AR441. **(B)** Syntokamides A-E. **(C)** EPI-001. **(D)** Niphatenone B.

3E10-AR441 (Figure 4A) is a bispecific antibody with the uncommon ability to enter the target cell and simultaneously bind two different targets (Goicochea et al., 2017). It is composed by two parts; one is a single-chain variable fragment of 3E10, an high-affinity DNA antibody, the other part is a single-chain variable fragment that target the AR NTD around aminoacid 441. The antibody binds wild-type, mutant and splice variants of AR and the authors also demonstrated the inhibition of cytoplasmic non-genomic related effects. Most of the existing antibodies employed in preclinical and clinical development and/or in current use target the extracellular domain of a protein or the cell membrane, because their large dimension do not allow them to pass into the cell. 3E10-AR441 has been developed to be a prototype antibody for cancer treatment.

### Sintokamides

Indonesian marine sponge *Dysidea* sp. was used to isolate the chlorinated peptides sintokamides A–E (Figure 4B), whose structures were investigated by spectroscopic and single X-ray diffraction analyses. Sintokamides, identified by Sadar et al. (2008), are the first natural products that specifically target AR NTD. In particular, sintokamide A resulted to be an inhibitor of AR-NTD in the androgen-sensitive LNCaP cells. Plus, it was found to be as effective as bicalutamide in blocking LNCaP proliferation after androgen stimulation. Recent *in vivo* studies also reported the ability of sintokamide A to selectively bind the AF1 portion of AR NTD, to inhibit forskolin-induced transactivation of AR NTD, to weaken both full-length AR and AR splice variants and to decrease the growth of CRPC xenografts, with a simultaneous reduction of PSA serum levels (Banuelos et al., 2016). To note, PSA is one of the most clinically validated indicator to monitor prostate cancer progression.

### EPI Compounds

EPI-compounds, isolated from a marine sponge named *Geodia lindgreni*, are structurally similar to Bisphenol A Diglycidic Ether (BADGE). BADGE derivatives from contaminated water were likely bioaccumulated in the collected sponge and cell-based assays were used to test 20 BADGE analogs in order to collect additional structure activity relationship for the pharmacophore. In particular, BADGE.HCl.H_2_O (also known as EPI-001; Figure 4C) emerged as the most promising compound, having the most potent activity (Andersen et al., 2010).

EPI-002 is one out of four stereoisomeres of racemic EPI-001, a chlorinated bisphenol compound with the ability to bind covalently the AR-NTD. EPI-506 is the oral prodrug derived from EPI-002 that underwent a promising phase 1 clinical trial (that included a phase 2 expansion) initiated in 2016 (NCT02606123). Unfortunately the trial, terminated in 2018, reported an excessive high pill burden (18 capsules/day) at the end of phase 1 and no other results are available, so further studies and ameliorations need to be undertaken.

### Niphatenones

Niphatenones had been originally isolated and identified in active extracts of dominica marine sponge *Niphates digitalis* (Meimetis et al., 2012). In particular, niphatenone B (Figure 4D) binds covalently to the AF1 region of AR NTD, blocking the proliferation of androgen-sensitive LNCaP cell line and making it a promising lead compound for drug design of similar AR NTD antagonists. Unfortunately, recent studies have discouraged further investigations on this class of compounds because alkylation experiments with glutathione adduct formation have indicated their high reactivity, with complementary low specificity (Banuelos et al., 2014). As a matter of fact, non-selective alkylators may potentially cause haptenation and toxicity. For this reason, they are not considered a feasible scaffold for further drug development.

## Conclusions

Many cellular responses to androgens and antiandrogens do not follow the canonical pathway driven by AR-mediated transcriptional activity. In fact, ligand-transformed AR acquires also the ability to bind molecular substrates which are present in the cytoplasm or in the inner layer of the cell membrane, resulting in a rapid intracellular kinase cascade.

Surgical and/or chemical androgen deprivation therapy is the main validated option for patients who relapse, providing some respite before the onset of the castration-resistant prostate cancer (CRPC) stage. CRPC patients, despite androgen deprivation, still have low levels of testosterone in serum (1–3 nM; Penning, 2014), that however largely comprise the range of non-genomic AR signaling pathways. Potent second-generation molecules, with different chemical structure and pharmacology, such as enzalutamide, apalutamide, or darolutamide, strongly bind AR-LBD and may reduce AR nuclear translocation, resulting in higher concentration of cytosolic AR enhancing/empowering the AR cytoplasmic action mechanisms. In particular, in epithelial PCa cells, AR NTD can potentially bind Src and PI3K (through p85α) triggering cell survival and proliferation pathways, through non-genomic AR-sustained mechanisms. This is an important escape pathway triggered by cytoplasmatic AR that should be taken into account by clinicians when considering the best therapeutic options for PCa patients. Targeting the AR NTD may not only reduce the recruitment of cytoplasmic binding partners, but could also be advantageous to target the AR splice variant still expressed in CRPC patients. Unfortunately, just few AR NTD inhibitors are at the moment both in preclinical and clinical development. Among them 3E10-AR441 and Sintokamide A seem to represent a promising starting point for the design and synthesis of new more selective molecules.

Epithelial-specific non-genomic AR functions have been lately described also in TME cells.

Recently, numerous authors have studied the TME composition as a prognostic factor in PCa (Galon et al., 2012; Halama et al., 2012; Kadota et al., 2015; Fridman et al., 2017). In addition, TME has also aroused substantial interest as a therapeutic target (Corn, 2012). Targeting different cell types representative of TME might represent an alternative, or coupled approach to standard therapies that barely target prostate epithelial cells (Karlou et al., 2010). In TME, the functionality of AR appears to acquire other important features, depending on the cell type considered. As previously reported, AR is diffusely expressed in TME, spanning from CAFs to immune cells and endothelial cells, but its expression tends to be lost, with the progression of the disease. In addition, it seems that the loss of stromal AR expression is related to the relapse of the disease and with poor prognosis (Penning, 2014), proposing stromal AR as a possible guardian in PCa disease.

Furthermore, stromal-epithelial crosstalk is considered to contribute to the acquisition of androgen resistance. In general, stromal cells produce various growth factors, independently from androgens, that are able to sustain PCa cells growth. Moreover, it was recently found that extracellular vesicles (EVs) isolated from PCa cell lines contains AR, and that it can be vehiculated to the nucleus of AR-null cells. In this “receiving”/“target” cells, then, it can bind the PSA promoter and form a classic transcription complex involving DNA pol II, resulting in enhanced proliferation (Read et al., 2017). In this context, non-genomic functions of the receptor were not investigated, but it would not be surprising if these kinds of effects were the first to occur. In the light of an EV-based diagnostic or therapeutic development, this suggests how AR could carry pro-tumorigenic information to TME cells and non-genomic pathways may represent one of the major outcomes.

In conclusion, targeting both genomic and non-genomic activity of AR could lead to the eradication of AR tumor dependency in PCa patients. Providing new combinatorial microenvironment-targeting strategies is urgently needed, focusing on the early stages of disease. Particularly challenging but fascinating is the idea to target specific activated stromal pathways to offer a personalized TME-based anticancer therapy in the next future.

## Author Contributions

AT and AZ conceived the idea for the research project, designed the review, and wrote the first draft of the manuscript. MC and MZ critically contributed to the work by describing the genomic role of AR in prostate cancer and provided valuable feedback on the first draft. All authors performed the literature review and all read and approved the final version of the work for submission.

### Conflict of Interest

The authors declare that the research was conducted in the absence of any commercial or financial relationships that could be construed as a potential conflict of interest.
